# Guidance for post-discharge care following acute kidney injury: an appropriateness ratings evaluation

**DOI:** 10.3399/bjgpopen20X101054

**Published:** 2020-06-17

**Authors:** Jung Yin Tsang, Jonathan Murray, Edward Kingdon, Charlie Tomson, Kyle Hallas, Stephen Campbell, Tom Blakeman

**Affiliations:** 1 National Institute for Health Research Collaboration for Leadership in Applied Health Research and Care (NIHR CLAHRC) Greater Manchester, Centre for Primary Care and Health Services Research, Institute of Population Health, University of Manchester, Manchester, UK; 2 NIHR Greater Manchester Patient Safety Translational Research Centre (PTSRC), University of Manchester, Manchester, UK; 3 Renal Unit, South Tees Hospitals NHS Foundation Trust, Middlesbrough, UK; 4 Academic Health Science Network for the North East and North Cumbria (AHSN NENC), Newcastle upon Tyne, UK; 5 Brighton & Sussex University Hospitals NHS Trust, Brighton, UK; 6 Kent Surrey Sussex Academic Health Science Network (KSS AHSN), Crawley, UK; 7 Department of Renal Medicine, Newcastle upon Tyne Hospitals NHS Foundation Trust, Newcastle upon Tyne, UK; 8 RCGP Clinical Champion for Kidney Care, Royal College of General Practitioners, London, UK

**Keywords:** acute kidney injury, heart failure, patient discharge, primary health care, general practice

## Abstract

**Background:**

Acute kidney injury (AKI) is associated with poor health outcomes, including increased mortality and rehospitalisation. National policy and patient safety drivers have targeted AKI as an example to ensure safer transitions of care.

**Aim:**

To establish guidance to promote high-quality transitions of care for adults following episodes of illness complicated by AKI.

**Design & setting:**

An appropriateness ratings evaluation was undertaken using the RAND/UCLA Appropriateness Method (RAM). The Royal College of General Practitioners (RCGP) AKI working group developed a range of clinical scenarios to help identify the necessary steps to be taken following discharge of a patient from secondary care into primary care in the UK.

**Method:**

A 10-person expert panel was convened to rate 819 clinical scenarios, testing the most appropriate time and action following hospital discharge. Specifically, the scenarios focused on determining the appropriateness and urgency for planning: an initial medication review; monitoring of kidney function; and assessment for albuminuria.

**Results:**

Taking no action (that is, no medication review; no kidney monitoring; or no albuminuria testing) was rated inappropriate in all cases. In most scenarios, there was consensus that both the initial medication review and kidney function monitoring should take place within 1–2 weeks or 1 month, depending on clinical context. However, patients with heart failure and poor kidney recovery were rated to require expedited review. There was consensus that assessment for albuminuria should take place at 3 months after discharge following AKI.

**Conclusion:**

Systems to support tailored and timely post-AKI discharge care are required, especially in high-risk populations, such as people with heart failure.

## How this fits in

Tackling the harms associated with AKI has been identified as a key priority to improve patient safety and health outcomes. RAND/UCLA consensus methodology was employed to navigate the challenge of overdiagnosis to maximise the utility of an AKI diagnosis, while minimising patient burden and reducing unnecessary clinical workload. High-quality transitions of care following AKI require patients to be offered a medication review and a blood test to monitor excretory kidney function, and a urine albumin-to-creatinine ratio (ACR) test, which is tailored according to clinical context. This study found that the strongest factors prompting an earlier clinical review were the presence of heart failure and poor kidney recovery (serum creatinine [SCr] >50% above pre-AKI baseline).

## Introduction

Ten years ago, the National Confidential Enquiry into Patient Outcome and Death (NCEPOD) report, *Adding Insult to Injury*, focused attention on the poor care surrounding AKI.^1^ Episodes of illness complicated by AKI are associated with: poor outcomes in terms of rehospitalisation; increased mortality (both short and long term); cardiovascular events; and development and progression of chronic kidney disease (CKD).^2^ AKI is common with an incidence rate in the UK reported to be 7.6 episodes per 100 hospital admissions.^3,4^ Within NHS England’s Patient Safety Domain, the Think Kidneys programme was established in 2014 to reduce the harm associated with AKI.^5^ Two subsequent national patient safety directives required implementation of a computerised alerting system within all NHS acute trusts and for all NHS providers to improve local systems and processes.^6^ Further national levers included a hospital-based Commissioning for Quality and Innovation (CQUIN) to improve discharge summaries and a recommendation by the National Institute for Health and Care Excellence (NICE) for general practices to generate a register of all people with a prior episode of AKI.^7,8^ However, to date, there remains limited evidence of post-discharge diagnostic coding of AKI in general practice.^9^ This appears to be multifactorial, including poor systems underpinning coding practice and the lack of recognition for the importance of coding AKI.^8^ Coding raises the awareness of the syndrome and has the potential to facilitate a more systematic approach to primary care management, as well as triggering underlying safety processes such as bloods recall and medication reviews.^6,8^

The last decade has seen a substantial increase in routine tests being undertaken in primary care, as well as a growing recognition of the harmful consequences of overdiagnosis.^10,11^ With concerns regarding overprescribing, there is a need for tailored care and a personalised approach to medicine.^12,13^ Therefore, maximising the clinical utility of AKI as a marker of poor patient outcomes must be balanced with a need to minimise the testing and treatment burden for patients and healthcare professionals. Evidence generated through the RCGP AKI case-note reviews revealed variable, ‘hit and miss’ discharge information leading to additional ‘digging’ work in general practice to manage the uncertainties created.^14^ It highlighted a need for tailored discharge summaries to determine the urgency of review, rather than producing generic instructions driven by tariff-based incentives.^15,16^

AKI is a clinical syndrome, not another long-term condition, and offers a lens to promote safer systems of care, particularly for people with multimorbidity.^17,18^ A consensus study was conducted to address gaps between work-as-imagined (guidance) and work-as-done (current routine practice).^19,20^ It sought to provide a platform to establish guidance for ensuring high-quality transitions of care in adults following an episode of illness complicated by AKI. To achieve this, the aim was to investigate and optimise the appropriateness and timeliness of post-discharge management actions in this heterogeneous patient group.

## Method

### Study design

The RAND/UCLA Appropriateness Method (RAM) was utilised for the present study. It is a recognised approach that systematically generates consensus through appropriateness ratings to help address clinical decision in areas where ‘robust evidence’ is lacking.^21–24^ It combines best available evidence with the shared knowledge of experts to provide a measure of the appropriateness of undertaking particular actions.^21–23^ It recognises that an appropriate response requires an understanding of the clinical context, which in this study related to the consideration of AKI within both primary and secondary care settings. For further information regarding the methodology, the development of guidance on the timeliness in response to AKI warning-stage test results for adults in primary care can be found in a 2016 evaluation by Blakeman *et al*, where a similar approach was adopted.^24^

### Developing clinical scenarios for appropriateness rating

An AKI working group (see Funding) was established through the RCGP to develop clinical scenarios for appropriateness rating, and built on the experience of the previous Think Kidneys working group.^24^ There was representation from primary and secondary care clinicians, as well as a methodologist with expertise in the RAM and patient safety in primary care. The working group collated and reviewed current evidence, including current guidance from NICE, CQUIN, and Think Kidneys for AKI, as well as recent international guidance.^7,25–27^ Through discussion of the key themes, priority was given to develop a range of scenarios to help identify necessary steps to be taken following the discharge of a patient from secondary care into primary care.

Scenarios were classified according to clinical characteristics (Table 1), including AKI severity (stage 1, 2, or 3), degree of kidney recovery at discharge (based on the baseline SCr value as determined by the NHS England algorithm^28^ for detecting AKI; with recovery defined at ≤25% above baseline, >25% and <50% above baseline, and ≥50% above baseline), and factors known to increase risk in the previous medical history.^26,29–31^ The approach taken recognised that AKI needs to be considered in the context of supporting care for people living with a range of complex health and social needs (that is, multimorbidity).^18,32^ Significant medical histories taken into account included: CKD;^29^ heart failure;^33,34^ heart failure with CKD;^35^ other significant cardiovascular risk factors (diabetes, hypertension, and established cardiovascular disease);^36–38^ markers of vulnerability (recurrent AKI,^39^ cancer treatment,^40^ sepsis,^39,41^ critical care admission,^42,43^ liver disease,^44^ low albumin,^45^ and/or chronic obstructive pulmonary disease);^39^ and other markers of frailty (those defined within NHS England's Toolkit for General Practice in Supporting Older People Living with Frailty).^46^ In terms of decision-making, the scenarios focused on determining the need and urgency for planning an initial medication review, as well as monitoring of kidney function and assessment for albuminuria (ACR) following hospital discharge.

**Table 1.** Key characteristics of categorisations of the clinical scenarios tested

**AKI severity**	**Kidney recovery**	**Clinical history**	**Management: next steps for rating**
AKI stage 1 AKI stage 2 AKI stage 3	Good (serum creatinine within ≤25% above baseline) Moderate (serum creatinine >25% and <50% above baseline) Poor (serum creatinine ≥50% above baseline)	Chronic kidney disease Chronic heart failure Chronic heart failure with chronic kidney disease Other significant cardiovascular risk factors Markers of vulnerability Markers of frailty	Determine appropriateness and timeliness in medication review Determine appropriateness and timeliness in kidney monitoring for serum creatinine Determine appropriateness and timeliness in kidney monitoring for proteinuria (urinary ACR)

ACR = albumin-to-creatinine ratio. AKI = acute kidney injury.

### Panel membership and rating

Panel members were purposively recruited to enable a balanced perspective specific to the healthcare professionals delivering routine care, aligned with RAM recommendations for a multidisciplinary approach.^21^ A 10-person panel was established comprising one nephrologist, one acute physician (with dual accreditation in nephrology), two AKI nurse specialists, and six GPs (two with an interest in heart failure, two with an interest in AKI, and two recruited through the RCGP overdiagnosis group).^47^ The panel was co-chaired by one researcher, who is an expert in the RAM, and a nephrologist, who is an expert in AKI, neither of whom contributed to the decision-making scores. Members of the AKI working group did not engage in panel rating but were present to facilitate the Round Two meeting (see below) and volunteer evidence for specific discussion points, including the rationale behind scenarios. Rating scales were as defined by established RAM.^21,24^

The RAND/UCLA process entails two rounds of ratings:

- Round One comprised panel members rating the clinical scenarios individually on an emailed spreadsheet. This was accompanied by an ‘instructions’ document and a ‘context’ document, which provided an overview of current literature and the rationale for the clinical characteristics chosen.
- Round Two comprised a 1-day, face-to-face meeting held in March 2019. Panel members were presented with summaries, including their own data from Round One, median values, and the spread of all panel members' ratings. This was used to stimulate discussion with a focus on areas of disagreement. Panel members then rated each scenario on their own individual-blinded rating sheets. Panel members were not required to reach consensus.

### Data entry and analysis

Data from Round Two were collated, validated, and entered by two independent members of support staff. Analysis then took place to determine the level of agreement within the panel for a proposed action in each clinical scenario. Possible ratings for each scenarios were on a scale from 1–9 and the analysis followed the definitions for agreement, disagreement, and equivocal as stated in standard RAM:^21^

- Agreement was defined by eight out of the 10 (80%) panel members rating the same three-point region on the nine-point integer scale (that is, 1–3, 4–6, 7–9). A proposed action was then categorised as an ‘appropriate’ next step if an agreement scenario was rated 7–9, with a rating of 1–3 defined to be an ‘inappropriate’ next step.
- Agreement with uncertain benefit is defined as 80% of panel members rating the same consecutive three-point region, but not 1–3 or 7–9.
- Disagreement was defined to exist where ≥30% of panel members rated a scenario in the 1–3 range and where ≥30% rated the same scenario in the 7–9 range on the nine-point integer scale.
- Ratings of clinical scenarios without consensus (either ‘agreement’ or ‘disagreement’) were considered as equivocal.

For scenarios with two timelines that reached agreement, the highest mean average was deemed to be the superior consensus based on the greater certainty of clinical priority.

## Results

Tables 2–5 summarise the results of 819 scenarios for Round Two ratings. There was agreement in 465 (56.8%) scenarios, with 24 (2.9%) rated with disagreement and 271 (33.1%) with an equivocal rating. An appropriate next step was identified in 214 (21.6%) scenarios. For all scenarios there was agreement that no action (that is, no medication review, no kidney monitoring, or no urine ACR) was inappropriate. Results are presented as medians to facilitate interpretation.

**Table 2.** Summary of round-two appropriateness ratings with definitions of consensus, *N* = 819.

**Appropriateness ratings**	***n* (%)**
Agreement (8/10 [80%] of panel members rating in the same three-point region)	465 (56.8%)
Appropriate (80% of panel members rating 7–9)	214 (26.1%)
Inappropriate (80% of panel members rating 1–3)	251 (30.6%)
Agreement with uncertain benefit (80% of panel members rating the same consecutive three-point region, but not 1–3 or 7–9)	59 (7.2%)
Disagreement (≥30% of scores in 1–3 and ≥30% in 7–9 for same scenario)	24 (2.9%)
Equivocal (ratings of clinical scenarios without consensus; that is, neither ‘agreement’ nor ‘disagreement’)	271 (33.1%)

**Table 3.** Summary of timeliness for performing the first post-discharge medication review.

**Medication review ratings**	**AKI Warning Stage 1**	**AKI Warning Stage 2**	**AKI Warning Stage 3**
Kidney recovery (SCr % above baseline)	≤ 25%	>25% & <50%	≥ 50%	≤ 25%	>25% & <50%	≥ 50%	≤ 25%	>25% & <50%	≥ 50%
	**Median (Rating**)	**Median (Rating**)	**Median (Rating**)	**Median (Rating**)	**Median (Rating**)	**Median (Rating**)	**Median (Rating**)	**Median (Rating**)	**Median (Rating**)
No past medical history	Not required	1.5 (I)	1 (I)	1 (I)	1 (I)	1 (I)	1 (I)	1 (I)	1 (I)	1 (I)
	Med r/v at 3 days	1.5 (I)	2 (I)	3.5 (E)	2 (I)	3 (E)	4.5 (E)	3 (I)	3.5 (E)	6.5 (D)
	Med r/v at 1–2 weeks	2.5 (I)	4 (E)	7 (E)	6 (D)	7.5 (U)	8 (A)	7.5 (E)	8 (E)	8 (E)
	Med r/v at 1 month	6.5 (E)	7 (U)	6.5 (U)	7 (E)	7 (U)	6.5 (E)	7 (U)	6.5 (U)	5.5 (E)
	Med r/v at 3 months	7 (E)	6 (E)	5.5 (E)	6.5 (E)	4.5 (E)	3.5 (E)	5 (E)	4 (E)	2.5 (E)
Chronic kidney disease	Not required	1 (I)	1 (I)	1 (I)	1 (I)	1 (I)	1 (I)	1 (I)	1 (I)	1 (I)
	Med r/v at 3 days	3 (I)	4 (E)	5.5 (E)	3 (E)	4 (E)	6.5 (E)	3 (E)	4 (E)	4 (E)
	Med r/v at 1–2 weeks	5.5 (E)	8 (E)	8 (A)	7 (E)	8 (A)	8 (A)	7.5 (E)	8.5 (A)	8.5 (A)
	Med r/v at 1 month	8 (A)	8 (A)	6.5 (E)	8 (A)	7 (A)*	5 (E)	7 (A)	7 (A)*	7 (A)*
	Med r/v at 3 months	5.5 (U)	3.5 (E)	3.5 (E)	5 (E)	3 (E)	2 (E)	4.5 (E)	3 (E)	3 (E)
Chronic heart failure	Not required	1 (I)	1 (I)	1 (I)	1 (I)	1 (I)	1 (I)	1 (I)	1 (I)	1 (I)
	Med r/v at 3 days	4.5 (U)	5 (U)	7 (U)	4 (E)	5.5 (U)	8 (A)*	5 (E)	6 (E)	9 (A)
	Med r/v at 1–2 weeks	8 (A)	8.5 (A)	9 (A)	8 (A)	8 (A)	9 (A)	9 (A)	8.5 (A)	8 (A)*
	Med r/v at 1 month	7 (U)	6 (E)	5.5 (E)	6 (U)	6 (E)	5 (E)	5.5 (U)	5 (E)	3.5 (E)
	Med r/v at 3 months	4 (E)	3 (E)	2.5 (I)	4 (E)	2.5 (E)	2 (I)	4 (E)	2 (I)	1.5 (I)
Chronic kidney disease & chronic heart failure	Not required	1 (I)	1 (I)	1 (I)	1 (I)	1 (I)	1 (I)	1 (I)	1 (I)	1 (I)
	Med r/v at 3 days	4.5 (E)	6 (U)	8 (A)*	5.5 (E)	6 (U)	8 (A)*	6 (E)	8 (A)*	9 (A)
	Med r/v at 1–2 weeks	8 (A)	9 (A)	9 (A)	9 (A)	9 (A)	9 (A)	9 (A)	9 (A)	8 (A)*
	Med r/v at 1 month	6 (E)	6.5 (E)	4.5 (D)	6 (E)	4.5 (E)	3.5 (D)	5.5 (E)	4 (E)	2.5 (E)
	Med r/v at 3 months	3 (E)	2.5 (E)	2 (I)	3 (E)	2 (I)	1.5 (I)	2.5 (E)	1.5 (I)	1 (I)
Other significant cardiovascular risk factors	Not required	1 (I)	1 (I)	1 (I)	1 (I)	1 (I)	1 (I)	1 (I)	1 (I)	1 (I)
	Med r/v at 3 days	2.5 (I)	3.5 (U)	5 (E)	3 (U)	4 (E)	6.5 (E)	4 (U)	4.5 (E)	7 (A)*
	Med r/v at 1–2 weeks	6 (U)	7.5 (A)*	8 (A)	7 (E)	8 (A)	9 (A)	8 (E)	9 (A)	9 (A)
	Med r/v at 1 month	8 (A)	8 (A)	6.5 (E)	8 (A)	7.5 (A)*	6 (E)	8 (A)	7.5 (A)*	5.5 (D)
	Med r/v at 3 months	6 (U)	5 (E)	3 (I)	4.5 (U)	3 (I)	2 (I)	5 (E)	2.5 (I)	1.5 (I)
Markers of vulnerability	Not required	1 (I)	1 (I)	1 (I)	1 (I)	1 (I)	1 (I)	1 (I)	1 (I)	1 (I)
	Med r/v at 3 days	3 (U)	4 (U)	6 (E)	3 (U)	4.5 (U)	6 (U)	4 (E)	5.5 (U)	8 (A)*
	Med r/v at 1–2 weeks	6.5 (E)	8 (A)	8.5 (A)	7 (E)	8 (A)	9 (A)	8 (A)*	9 (A)	8 (A)
	Med r/v at 1 month	8 (A)	7 (A)*	7 (E)	8 (A)	7.5 (A)*	6.5 (E)	8 (A)	7.5 (E)	6 (D)
	Med r/v at 3 months	5 (E)	4 (E)	3.5 (E)	4.5 (E)	3.5 (E)	3 (I)	4 (E)	3 (I)	1.5 (I)
Other markers of frailty	Not required	1 (I)	1 (I)	1 (I)	1 (I)	1 (I)	1 (I)	1 (I)	1 (I)	1 (I)
	Med r/v at 3 days	3 (U)	4 (U)	6 (E)	3 (I)	4 (U)	6.5 (E)	3 (E)	5 (E)	8 (A)*
	Med r/v at 1–2 weeks	7 (U)	7.5 (A)	8 (A)	7 (E)	8 (A)	9 (A)	8 (E)	9 (A)	8.5 (A)
	Med r/v at 1 month	8 (A)	7.5 (U)	6.5 (E)	8 (A)	7 (A)*	6 (E)	8 (A)	7 (A)*	5.5 (D)
	Med r/v at 3 months	5.5 (D)	5 (E)	3 (E)	5 (E)	3 (U)	2 (I)	4.5 (E)	3 (I)	2 (I)

The colour of each cell represents the result with the highest consensus. Grey cells are given where consensus was not reached. A = appropriate. A* = appropriate but with a lower average certainty. AKI = acute kidney injury. D = disagreement. E = equivocal. I = inappropriate. Med r/v = medication review. SCr = serum creatinine. U = agreement with uncertain benefit.****

**Table 4.** Summary of timeliness for performing the first post-discharge serum creatinine test.

**Serum creatinine monitoring ratings**	**AKI Warning Stage 1**	**AKI Warning Stage 2**	**AKI Warning Stage 3**
Kidney recovery (SCr % above baseline)	≤ 25%	>25% & <50%	≥ 50%	≤ 25%	>25% & <50%	≥ 50%	≤ 25%	>25% & <50%	≥ 50%
	**Median (Rating**)	**Median (Rating**)	**Median (Rating**)	**Median (Rating**)	**Median (Rating**)	**Median (Rating**)	**Median (Rating**)	**Median (Rating**)	**Median (Rating**)
No past medical history	Not required	1 (I)	1 (I)	1 (I)	1 (I)	1 (I)	1 (I)	1 (I)	1 (I)	1 (I)
	SCr at 3 days	1 (I)	1.5 (I)	3 (E)	1.5 (I)	2.5 (I)	3.5 (E)	2 (I)	3.5 (E)	5.5 (E)
	SCr at 1–2 weeks	2.5 (I)	3.5 (D)	7 (U)	2.5 (E)	6 (D)	8 (A)	4 (E)	7.5 (A)	8 (A)
	SCr at 1 month	5 (E)	7.5 (E)	8 (A)	7.5 (E)	8.5 (A)	7.5 (E)	7.5 (E)	8 (A)	6.5 (E)
	SCr at 3 months	8 (A)	7 (E)	5.5 (D)	7.5 (E)	7 (E)	4 (D)	7.5 (E)	6 (E)	4 (E)
Chronic kidney disease	Not required	1 (I)	1 (I)	1 (I)	1 (I)	1 (I)	1 (I)	1 (I)	1 (I)	1 (I)
	SCr at 3 days	3 (I)	3.5 (E)	4.5 (E)	2.5 (I)	3 (E)	6 (E)	3 (I)	4 (E)	6 (E)
	SCr at 1–2 weeks	4.5 (D)	6.5 (U)	7 (E)	5.5 (E)	8 (A)	8 (A)	7 (E)	8 (A)	9 (A)
	SCr at 1 month	8 (A)	8 (A)	8 (E)	8 (A)	8 (A)*	7.5 (E)	8 (A)	7.5 (E)	6 (E)
	SCr at 3 months	6.5 (E)	6 (D)	5 (D)	6.5 (E)	6 (E)	3.5 (E)	5 (D)	3 (E)	1.5 (E)
Chronic heart failure	Not required	1 (I)	1 (I)	1 (I)	1 (I)	1 (I)	1 (I)	1 (I)	1 (I)	1 (I)
	SCr at 3 days	2.5 (I)	4 (E)	5 (U)	3.5 (U)	4.5 (U)	6 (E)	3.5 (U)	6 (U)	7 (A)*
	SCr at 1–2 weeks	7 (U)	8 (A)	9 (A)	7.5 (A)*	8 (A)	9 (A)	7.5 (A)	8 (A)	8 (A)
	SCr at 1 month	8 (A)	7.5 (A)*	6 (E)	8 (A)	7 (E)	4.5 (E)	7.5 (U)	6.5 (E)	3.5 (E)
	SCr at 3 months	5 (D)	3.5 (E)	2.5 (I)	4.5 (E)	3 (E)	1.5 (I)	4.5 (E)	2 (I)	1.5 (I)
Chronic kidney disease & chronic heart failure	Not required	1 (I)	1 (I)	1 (I)	1 (I)	1 (I)	1 (I)	1 (I)	1 (I)	1 (I)
	SCr at 3 days	3.5 (E)	5 (U)	6 (U)	3 (E)	4.5 (U)	7 (U)	5 (E)	6 (U)	8 (A)*
	SCr at 1–2 weeks	7.5 (A)*	8 (A)	9 (A)	8 (A)	8 (A)	9 (A)	8 (A)	9 (A)	8.5 (A)
	SCr at 1 month	7.5 (A)	7 (E)	7 (E)	7.5 (E)	6.5 (E)	4 (D)	7.5 (E)	4.5 (E)	3 (E)
	SCr at 3 months	3 (E)	3 (E)	2.5 (I)	2.5 (E)	2.5 (E)	1.5 (I)	2.5 (E)	2.5 (I)	1.5 (I)
Other significant cardiovascular risk factors	Not required	1 (I)	1 (I)	1 (I)	1 (I)	1 (I)	1 (I)	1 (I)	1 (I)	1 (I)
	SCr at 3 days	2 (I)	3 (U)	4 (U)	3 (E)	3 (E)	4.5 (E)	3 (U)	4 (E)	6 (E)
	SCr at 1–2 weeks	4.5 (E)	7 (U)	8 (A)	5.5 (E)	8 (A)	8.5 (A)	7 (A)*	8 (A)	8 (A)
	SCr at 1 month	7.5 (A)	8 (A)	7.5 (A)*	8 (A)	7 (A)*	7 (U)	8.5 (A)	7 (U)	6.5 (E)
	SCr at 3 months	5.5 (E)	4.5 (E)	3.5 (E)	4.5 (E)	3.5 (E)	3 (I)	4.5 (E)	2.5 (E)	2 (I)
Markers of vulnerability	Not required	1 (I)	1 (I)	1 (I)	1 (I)	1 (I)	1 (I)	1 (I)	1 (I)	1 (I)
	SCr at 3 days	3 (I)	3.5 (E)	4.5 (U)	2.5 (I)	3.5 (E)	4.5 (E)	3 (E)	4 (E)	6 (E)
	SCr at 1–2 weeks	5 (E)	7 (U)	8 (A)	5 (E)	8 (A)	8.5 (A)	7 (E)	8 (A)	8.5 (A)
	SCr at 1 month	8 (A)	8 (A)	7 (A)*	8 (A)	7.5 (A)*	7.5 (A)*	8 (A)	7 (A)*	7 (E)
	SCr at 3 months	6 (U)	5.5 (E)	4.5 (E)	5.5 (E)	4 (E)	2.5 (E)	5 (D)	3.5 (E)	2 (I)
Other markers of frailty	Not required	1 (I)	1 (I)	1 (I)	1 (I)	1 (I)	1 (I)	1 (I)	1 (I)	1 (I)
	SCr at 3 days	2.5 (I)	3 (U)	3 (U)	2 (I)	3 (E)	4 (E)	3 (I)	4 (E)	6 (E)
	SCr at 1–2 weeks	4 (D)	7 (E)	8 (A)	5.5 (E)	8 (A)	9 (A)	7 (E)	8 (A)	9 (A)
	SCr at 1 month	8 (A)	8 (A)	7 (A)*	8 (A)	8 (A)*	7.5 (A)*	8 (A)	7 (A)*	6.5 (E)
	SCr at 3 months	6.5 (E)	5 (E)	3.5 (E)	5.5 (E)	4 (E)	2 (I)	4.5 (E)	3 (E)	2 (I)

The colour of each cell represents the result with the highest consensus. Grey cells are given where consensus was not reached. A = appropriate. A* = appropriate but with a lower average certainty. AKI = acute kidney injury. D = disagreement. E = equivocal. I = inappropriate. SCr = serum creatinine. U = agreement with uncertain benefit.**

**Table 5.** Summary of timeliness for performing the first post-discharge urine albumin-to-creatinine ratio.

**Urine ACR ratings**	**AKI Warning Stage 1**	**AKI Warning Stage 2**	**AKI Warning Stage 3**
Kidney recovery (SCr % above baseline)	≤ 25%	>25% & <50%	≥ 50%	≤ 25%	>25% & <50%	≥ 50%	≤ 25%	>25% & <50%	≥ 50%
	**Median (Rating**)	**Median (Rating**)	**Median (Rating**)	**Median (Rating**)	**Median (Rating**)	**Median (Rating**)	**Median (Rating**)	**Median (Rating**)	**Median (Rating**)
No past medical history	Not required	1 (I)	1 (I)	1 (I)	1 (I)	1 (I)	1 (I)	1 (I)	1 (I)	1 (I)
	UACR at 1 Month	2.5 (I)	2 (I)	2 (I)	2 (E)	2 (E)	2 (E)	2 (E)	2 (E)	2 (E)
	UACR at 3 Months	9 (A)	9 (A)	9 (A)	9 (A)	9 (A)	9 (A)	9 (A)	9 (A)	9 (A)
Chronic kidney disease	Not required	1 (I)	1 (I)	1 (I)	1 (I)	1 (I)	1 (I)	1 (I)	1 (I)	1 (I)
	UACR at 1 Month	2.5 (E)	2.5 (E)	2.5 (E)	2.5 (E)	2.5 (E)	2.5 (E)	2.5 (E)	2.5 (E)	3 (E)
	UACR at 3 Months	9 (A)	9 (A)	9 (A)	9 (A)	9 (A)	9 (A)	9 (A)	9 (A)	9 (A)
Chronic heart failure	Not required	1 (I)	1 (I)	1 (I)	1 (I)	1 (I)	1 (I)	1 (I)	1 (I)	1 (I)
	UACR at 1 Month	3.5 (E)	3.5 (E)	3.5 (E)	3.5 (E)	3.5 (E)	3.5 (E)	3.5 (E)	3.5 (E)	3.5 (E)
	UACR at 3 Months	9 (A)	9 (A)	9 (A)	9 (A)	9 (A)	9 (A)	9 (A)	9 (A)	9 (A)
Chronic kidney disease & chronic heart failure	Not required	1 (I)	1 (I)	1 (I)	1 (I)	1 (I)	1 (I)	1 (I)	1 (I)	1 (I)
	UACR at 1 Month	3.5 (E)	3.5 (E)	3.5 (E)	3.5 (E)	3.5 (E)	3.5 (E)	3.5 (E)	3.5 (E)	3.5 (E)
	UACR at 3 Months	9 (A)	9 (A)	9 (A)	9 (A)	9 (A)	9 (A)	9 (A)	9 (A)	9 (A)
Other significant cardiovascular risk factors	Not required	1 (I)	1 (I)	1 (I)	1 (I)	1 (I)	1 (I)	1 (I)	1 (I)	1 (I)
	UACR at 1 Month	4 (E)	4 (E)	4 (E)	4 (E)	4 (E)	4 (D)	4 (E)	4 (D)	4 (D)
	UACR at 3 Months	9 (A)	9 (A)	9 (A)	9 (A)	9 (A)	9 (A)	9 (A)	9 (A)	9 (A)
Markers of vulnerability	Not required	1 (I)	1 (I)	1 (I)	1 (I)	1 (I)	1 (I)	1 (I)	1 (I)	1 (I)
	UACR at 1 Month	3 (E)	3 (E)	3 (E)	3 (E)	3 (E)	3 (E)	3 (E)	3 (E)	3 (E)
	UACR at 3 Months	9 (A)	9 (A)	9 (A)	9 (A)	9 (A)	9 (A)	9 (A)	9 (A)	9 (A)
Other markers of frailty	Not required	1 (I)	1 (I)	1 (I)	1 (I)	1 (I)	1 (I)	1 (I)	1 (I)	1 (I)
	UACR at 1 Month	2 (E)	2 (E)	2 (E)	2 (E)	2 (E)	2 (I)	2 (E)	2 (E)	2 (E)
	UACR at 3 Months	9 (A)	9 (A)	9 (A)	9 (A)	9 (A)	9 (A)	9 (A)	9 (A)	9 (A)

In all urine albumin-to-creatinine ratio scenarios, the highest consensus was to perform the first test at 3 months (all cells coloured green to reflect highest consensus). A = appropriate. AKI = acute kidney injury. D = disagreement. E = equivocal. I = inappropriate. SCr = serum creatinine. U = agreement with uncertain benefit. UACR = urine albumin-to-creatinine ratio.

### Medication review

Table 3 outlines the ratings for considering the appropriateness and timeliness of a medication review post-discharge following AKI. Those with an agreement regarding an appropriate next step have been colour-coded in the table. There were only two scenarios warranting medication review within 3 days of hospital discharge (shown in red): heart failure combined with AKI stage 3 and poor kidney recovery (that is, SCr >50% above baseline) without pre-existing CKD; and the same combination of factors with pre-existing CKD. Heart failure was the most important factor prompting an earlier review, with all combinations (AKI stage and any degree of kidney recovery) requiring a medication review within 1–2 weeks (shown in yellow). In the presence of other risk factors, panellists generally rated scenarios with moderate to poor kidney recovery (SCr >25% and <50% above baseline) to require a medication review within 1–2 weeks (shown in yellow). Scenarios with good kidney recovery (SCr ≤25% above baseline), irrespective of AKI stage required medication review at 1 month (shown in light green). Consensus was not reached in several scenarios (shown in grey) describing patients without significant risk factors, although trends can be observed. The panel did not reach agreement on the choice between 1–2 weeks versus 1 month post-discharge for AKI stages 2 and 3, and between 1 month versus 3 months for AKI stage 1.

### Blood test monitoring

Table 4 outlines the ratings for considering the appropriateness and timeliness of repeating a SCr blood test post-discharge following AKI. There was generally good correlation between the timeliness for medication review and kidney monitoring. There were no scenarios that required a blood test within 3 days. Again, heart failure was the most important factor prompting an earlier review, with all scenarios requiring repeat blood tests at 1–2 weeks (shown in yellow) except when there was good kidney recovery from AKI stage 1. For all other risk factors, there was consensus that first repeat blood tests post-discharge should generally be performed at 1–2 weeks (shown in yellow) for patients with moderate to poor kidney recovery (SCr >25% and <50% above baseline) and at 1 month (shown in light green) if there was good kidney recovery (SCr ≤25% above baseline). The only exception was if patients had any significant medical history (apart from heart failure), AKI stage 1 and moderate kidney recovery (SCr >25% and <50% above baseline), where consensus recommended a repeat blood test at 1 month also (shown in light green). The only scenario where the first repeat blood test was deemed appropriate at 3 months (shown in dark green), was patients with no significant medical history, AKI stage 1, and good kidney recovery.

### Urine albumin-to-creatinine ratio

Table 5 shows the urine ACR results panel ratings for urine ACR testing post-discharge. In all scenarios, monitoring at 3 months was deemed the only appropriate action. This was irrespective of any medical history, any AKI stage, or any degree of kidney recovery. Median scores for monitoring at 1 month was no greater than 4 for any scenario.

## Discussion

### Summary

**Figure 1.**
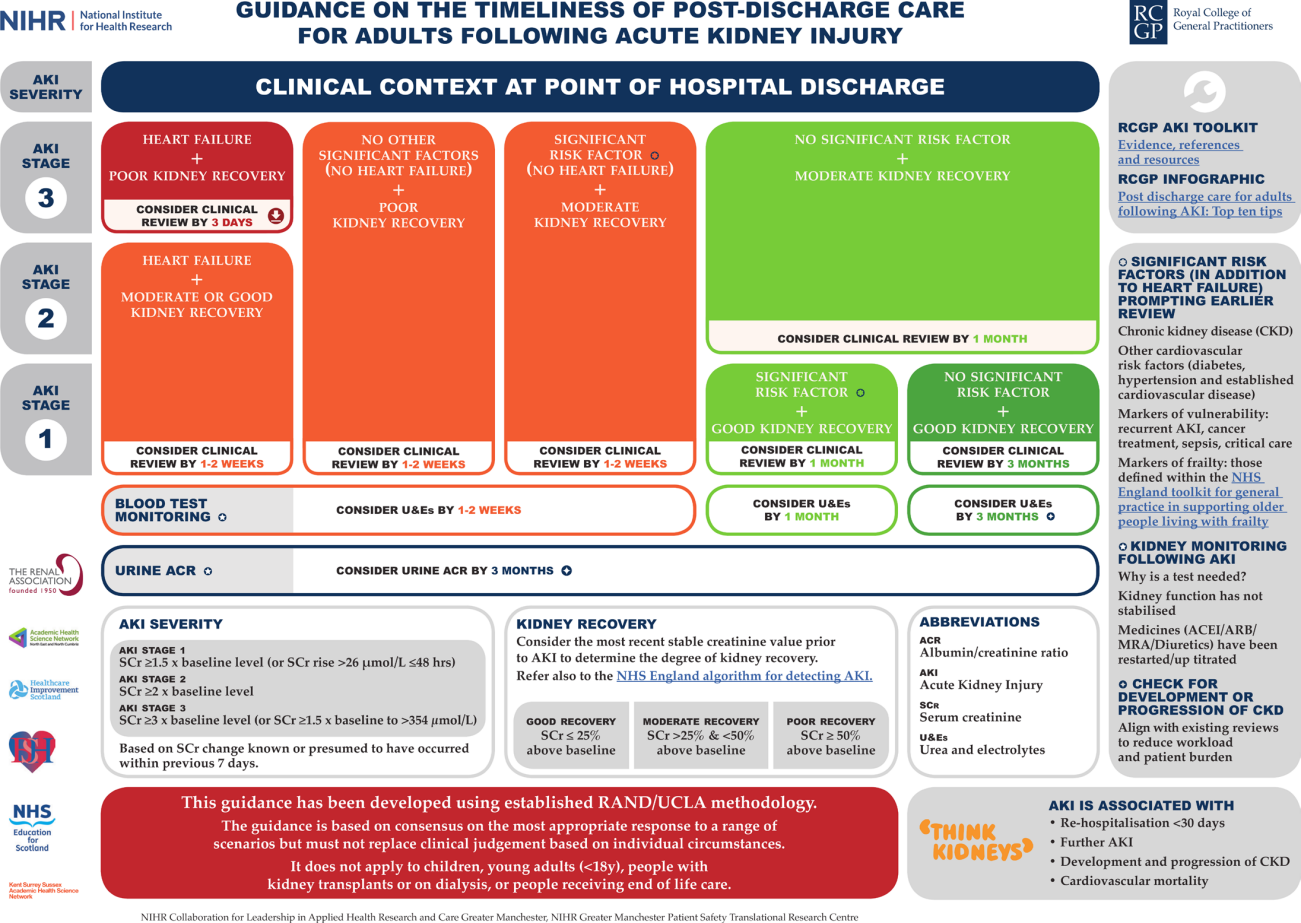
Guidance on the timeliness of post-discharge care for adults following acute kidney injury (AKI).

The findings from the study have informed the development of RCGP guidance to support post-discharge care for people who have had a hospital admission complicated by AKI (Figure 1). This guidance seeks to ensure tailored and timely post-hospital AKI care, which is based on individual clinical context. The strongest factors prompting earlier clinical review were the presence of heart failure and poor kidney recovery.

### Strengths and limitations

The strength in the study's approach lies in the practical, real-world nature of the RAM method, tailored to individual need and taking into account decision-making grounded in everyday practice.^21^ Presently, there is widespread criticism that current medicine perpetuates the problem of over-testing and overdiagnosis, calling for studies and guidelines to adopt a more holistic people-centred approach that takes these challenges into account.^18,48,49^ In order to navigate the challenge of maximising utility but minimising burden of AKI, rigorous RAM was used with representation from secondary and primary care. This included recruitment through the RCGP overdiagnosis working group.^21,47^ The RAM can be considered a modified Delphi method, and carries advantages over the Dephi and other consensus methodology, such as nominal group technique or the National Institutes of Health Consensus Development Conference, in that it incorporates evidence into the discussion and requires a separate expert group to be established to generate scenarios.^50^

Owing to the pragmatic nature of the methodology, only a limited number of scenarios and risk factors could be tested. With every additional risk factor tested, the number of scenarios increased exponentially, increasing risk of fatigue impacting on the rating process. Number of prior hospitalisations, length of stay, and destination at discharge (for example, care home) were not evaluated but may be relevant.^51,52^ However, it is unlikely that these factors will have affected the results significantly, since similar results were observed through testing several factors for both markers of vulnerability and frailty. Bias may also have been introduced through categories being imposed, since the study was not designed to explore bundling categories together. For example, the provision of 3 days as the earliest review point (intentionally selected to reflect the minimum timeframe for patients discharged over the weekend) may lead to greater tendency to opt for 1–2 weeks, than may have occurred if 1–2 weeks had been the earliest option available.

### Comparison with existing literature

The findings highlight that heart failure was found to be the most critical factor prompting earlier clinical review following AKI. The findings recognise that a high proportion of hospital readmissions following AKI are owing to pulmonary oedema or decompensated heart failure.^52,53^ Resonating with other studies conducted in North America, Sawhney *et al* analysed a large Scottish database and found that up to one in four readmissions after AKI were related to acute pulmonary oedema, which could conceivably be prevented by timely reintroduction of drugs stopped during admission, for example, diuretics and angiotensin converting enzyme (ACE) inhibitors.^39,52,54^

The results indicate that post-AKI care needs to be aligned with, and built on, evidence underpinning the management of individuals with heart failure. Recent national guidance has been published to support clinicians on how to respond to changes in kidney function during treatment of heart failure.^55^ Although worsening kidney function during treatment of heart failure identifies a group with a poor prognosis, there is strong evidence that this relationship is not directly causal.^55^ In many situations, the change in kidney function is caused by haemodynamic alterations, affecting glomerular filtration but without evidence of tubular injury.^56–58^ The national guidance emphasises that clinical assessment is paramount and that treatment of congestion should be prioritised.^55^ It highlights that *‘* *achieving clinical euvolaemia is a* *fundamental* *goal to improve symptoms and* *improve* *outcome* *’*, and that if a patient is improving, then a decline in kidney function is of secondary importance.^55^

There was strong consensus that urine ACR should be assessed at 3 months to check for persistent renal injury and progression of CKD following AKI. This aligns with NICE guidance that recommends testing for at least 2–3 years even if SCr has returned to baseline.^29^ Patients require time for renal recovery to baseline function before concluding that persistent damage has occurred and 3 months is consistent with the literature for this period.^31^ Our guidance also supports recent evidence highlighting the importance of urine ACR as a factor for risk-stratification following AKI and recommends more extensive routine testing.^59^ However, there appears to be widespread reticence, with the national CKD audit revealing that there is limited testing of urine ACR, especially outside of patients with diabetes.^60^

### Implications for practice

The results on post-discharge actions call for a drive to ensure discharge summaries arrive on time from secondary to primary care. Electronic records are improving the efficiency and timeliness of this solitary communication method, yet there remains widespread variation worldwide.^61^ Many patients experience a gap in care rather than a transition as it is frequently unclear who is responsible for certain parts of their discharge care.^62,63^ The present study's consensus findings indicate that patients with heart failure in conjunction with AKI stage 3 and poor kidney recovery need to be reviewed within 3 days, and this could be more effective if coordinated from secondary care.

Incomplete recovery of kidney function after AKI predicts poorer long-term outcomes even in milder cases of AKI.^26,30,64,65^ Peak AKI stage is likely to be readily available to discharge summary authors; however, the baseline creatinine, which is necessary to determine the completeness of recovery, may not be obvious to either the discharge summary author or recipient in general practice. There may be a need for pathology labs or discharge summary platforms to automate assignment of a kidney recovery category. However, it is also recognised that this should not be at the detriment of discharge planning that takes into account a person’s broader clinical and social circumstance.^66^ SCr needs to be interpreted carefully owing to the effect of critical illness on muscle mass, and this interaction is likely to complicate assessment of kidney recovery following AKI.^67–69^

Through the RCGP, the authors' research and approach to quality improvement builds on an international evidence base by establishing national recommendations for the appropriateness and timeliness of post-discharge actions following AKI in the UK.^26^ Through a pragmatic approach consensus, guidance has been established that seeks to navigate the challenges of too much medicine.^13^ While aiming to promote post-discharge care that aligns patient safety with routine practice, this study highlights that the presence of heart failure and poor kidney recovery should prompt early clinical review following AKI episodes in hospital.
